# G﻿lobal phylogeography of ridley sea turtles (*Lepidochelys* spp.): evolution, demography, connectivity, and conservation

**DOI:** 10.1007/s10592-022-01465-3

**Published:** 2022-08-29

**Authors:** Sibelle Torres Vilaça, Anelise Torres Hahn, Eugenia Naro-Maciel, F. Alberto Abreu-Grobois, Brian W. Bowen, Jaqueline C. Castilhos, Claudio Ciofi, Nancy N. FitzSimmons, Michael P. Jensen, Angela Formia, Colin J. Limpus, Chiara Natali, Luciano S. Soares, Benoit de Thoisy, Scott D. Whiting, Sandro L. Bonatto

**Affiliations:** 1 Department of Life Sciences and Biotechnology, University of Ferrara, Ferrara, Italy; 2 Escola de Ciências da Saúde e da Vida, Pontifícia Universidade Católica do Rio Grande do Sul, Av. Ipiranga, 668, 90619-900 Porto Alegre, RS Brazil; 3 Liberal Studies, New York University, 726 Broadway, New York, USA; 4 Instituto de Ciencias del Mar y Limnología, Unidad Académica Mazatlán, Universidad Nacional Autónoma de México, Sinaloa, México; 5 Hawaiʻi Institute of Marine Biology, University of Hawaiʻi, Kaneohe, Hawaiʻi USA; 6 Fundação Projeto Tamar, Sergipe, Brazil; 7 Department of Biology, University of Florence, Sesto Fiorentino (FI), Florence, Italy; 8 Australian Rivers Institute, Griffith University, South East Queensland, Australia; 9 Department of Chemistry and Bioscience, Aalborg University, Aalborg, Denmark; 10 Gulf of Guinea Sea Turtle Program, Wildlife Conservation Society, Libreville, Gabon; 11 Department of Environment and Resource Management, Brisbane, QLD Australia; 12 Archie Carr Center for Sea Turtle Research, University of Florida, Gainesville, FL USA; 13 Florida Fish and Wildlife Conservation Commission, Fish and Wildlife Research Institute, Saint Petersburg, FL USA; 14 Kwata NGO, Cayenne, French Guiana; 15 Department of Biodiversity, Conservation and Attractions, Kensington, WA Australia

**Keywords:** Population structure, mtDNA control region, Microsatellites, Demographic change, Effective population size, Marine conservation, Marine turtle

## Abstract

Globally distributed marine taxa are well suited for investigations of biogeographic impacts on genetic diversity, connectivity, and population demography. The sea turtle genus *Lepidochelys* includes the wide-ranging and abundant olive ridley (*L. olivacea*), and the geographically restricted and ‘Critically Endangered’ Kemp’s ridley (*L. kempii*). To investigate their historical biogeography, we analyzed a large dataset of mitochondrial DNA (mtDNA) sequences from olive (n = 943) and Kemp’s (n = 287) ridleys, and genotyped 15 nuclear microsatellite loci in a global sample of olive ridleys (n *=* 285). We found that the ridley species split ~ 7.5 million years ago, before the Panama Isthmus closure. The most ancient mitochondrial olive ridley lineage, located in the Indian Ocean, was dated to ~ 2.2 Mya. Both mitochondrial and nuclear markers revealed significant structure for olive ridleys between Atlantic (ATL), East Pacific (EP), and Indo-West Pacific (IWP) areas. However, the divergence of mtDNA clades was very recent (< 1 Mya) with low within- clade diversity, supporting a recurrent extinction-recolonization model for these ocean regions. All data showed that ATL and IWP groups were more closely related than those in the EP, with mtDNA data supporting recent recolonization of the ATL from the IWP. Individual olive ridley dispersal between the ATL, EP, and IN/IWP could be interpreted as more male- than female-biased, and genetic diversity was lowest in the Atlantic Ocean. All populations showed signs of recent expansion, and estimated time frames were concordant with their recent colonization history. Investigating species abundance and distribution changes over time is central to evolutionary biology, and this study provides a historical biogeographic context for marine vertebrate conservation and management.

**Supplementary Information:**

The online version contains supplementary material available at 10.1007/s10592-022-01465-3.

## Introduction

Understanding the impacts of past biogeographic processes on recent demographic and genetic patterns is fundamental to the fields of evolutionary and conservation biology. For instance, historical climate cycles throughout glacial and interglacial periods significantly influenced temporal and spatial distributions of extant taxa (Dynesius and Jansson 2000). Further, the relationship between demographic change, patterns of genetic diversity, and population connectivity is key to inferring evolutionary potential (Frankham et al. 2017). In light of the planet’s current biodiversity and climate crises (IPBES 2019; IPCC 2022), this historical knowledge is becoming ever more important for inferring how species may respond to contemporary threats and for informing conservation strategies (Bowen and Karl 2007; Naro-Maciel et al. 2014; Vilaça et al. 2014; Reid et al. 2019). Genetic analysis of globally distributed marine species, including sister taxa differentially impacted by historical circumstances, can illuminate critical aspects of biogeography by recovering past global and local oceanic processes that influenced genetic diversity and population dynamics.

Despite their ancient evolutionary history, sea turtles (superfamily Chelonioidea) are currently under threat of extinction. Six species are classified globally from ‘Vulnerable’ to ‘Critically Endangered,‘ and the seventh is considered ‘Data Deficient’ (IUCN 2022). Extant chelonioid genera are monotypic except for the two species of ridley turtles (*Lepidochelys* spp.). The olive ridley (*L. olivacea*) is listed as ‘Vulnerable’ and is the most abundant sea turtle, occupying all tropical and temperate oceans except the North Atlantic (Abreu-Grobois and Plotkin 2008). In contrast, the Critically Endangered Kemp’s ridley (*L. kempii*) is the rarest marine turtle, with a nesting distribution primarily restricted to the Gulf of Mexico. Most Kemp’s ridley nests occur in the vicinity of Rancho Nuevo, Mexico, and Padre Island National Sea Shore, USA, where olive ridleys do not generally nest (Abreu-Grobois and Plotkin 2008; SEMARNAT 2018; Wibbels and Bevan 2019). The main threats faced by both ridley species are egg harvest, coastal development, pollution, climate change, ingestion of plastics, and fisheries impacts (including direct take, bycatch, and entanglement in abandoned fishery equipment or “ghost nets”) (Abreu-Grobois and Plotkin 2008; Wibbels and Bevan 2019; Stelfox et al. 2019, 2020). Both species exhibit characteristic nesting behaviors: they can either nest solitarily, or synchronously in mass nesting events known as “arribadas” in which numerous females lay their eggs after gathering in nearshore waters (Bernardo and Plotkin 2007). Some of the arribada rookeries in the Eastern Pacific (Costa Rica, Mexico) and the East Coast of India (Bowen et al. 1997; Shanker et al. 2004, 2021) have between 200,000 and 2,000,0000 nests per year, and their sheer sizes drive olive ridley population trends at regional levels.

The two ridley species’ taxonomic distinctiveness and evolutionary history were obscured by their morphological similarity (Pritchard 1969) until genetic analyses validated their species status (Bowen et al. 1991; Naro-Maciel et al. 2008). An early hypothesis posited allopatric speciation caused by the closure of the Panama Isthmus about 3–5 million years ago (Mya) (Pritchard 1969). In this scenario, a putative ancestral ridley species would have been split by the vicariant barrier such that Kemp’s ridleys would have evolved in the Atlantic and olive ridleys in the East Pacific. The olive ridley range would then have expanded through the Eastern Pacific into the Indo-Pacific during the late Pliocene and Pleistocene, and more recently into the Atlantic via the Cape of Good Hope (Pritchard 1967; Bowen et al. 1997). In an alternative hypothesis, Shanker et al. (2004) countered that the ancestral olive ridleys instead radiated out from the Indian Ocean into both the Pacific and Atlantic, rather than from the vicinity of Panama. They showed that the most divergent olive ridley haplotypes, those most closely related to the Kemp’s, occurred in the Indian Ocean. This finding supported the hypothesis that during glacial periods, Indo-Pacific waters warmed by the Indonesian Sea Way (Cane and Molnar 2001) provided a stable environment, which stood in contrast to the disruptions of the rising Isthmus, and served as a refuge and a source for later colonizations (Shanker et al. 2004).

Ridley populations have endured fluctuations throughout their evolutionary history. Following the Last Glacial Maximum (LGM) 26,000–19,000 years before present (YBP) and the Holocene onset ~ 11,700 YBP, sea-level rise would have flooded many sea turtle rookeries but allowed colonization of new nesting areas. Indeed, synchronous population expansion after the LGM was revealed in most global sea turtle mtDNA lineages, including ridleys (Reid et al. 2019). More recently, and relevant to conservation efforts, a putatively anthropogenic bottleneck was reported to have reduced Atlantic olive ridley genetic diversity in the past 2,000 years (Plot et al. 2012).

With a new and substantially expanded data set of geographical genetic patterns, this study revisited and updated these hypotheses on the population history, phylogeography, and distribution of ridley sea turtles, as well as their global management units. Previous research relied on geographically restricted sampling, mitochondrial sequences, and ensuing limited inference power (Bowen et al. 1997; Jensen et al. 2013; Campista León et al. 2019; Work et al. 2019; Adnyana et al. 2020; Silver-Gorges et al. 2020; Stelfox et al. 2020). Our goals were therefore to (i) investigate current geographic patterns of genetic diversity in ridley populations around the world, (ii) determine genetic relationships between populations and oceanic regions, (iii) explore changes in temporal and spatial population distributions, (iv) infer divergence and differentiation times between populations and the two ridley species, and (v) compare ridley phylogeographical patterns to those of other sea turtle taxa.

## Materials and methods

### Sampling and DNA extraction

**Fig. 1:**
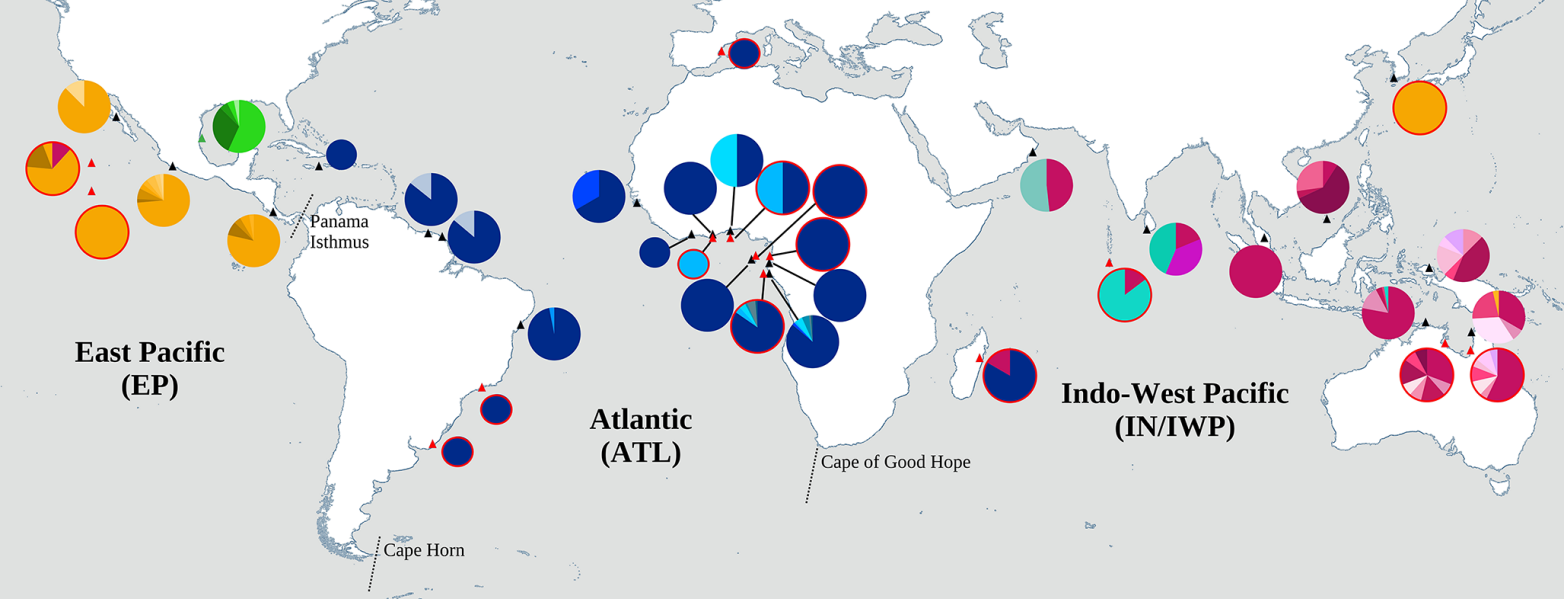
Map showing mtDNA control region haplotype frequencies per sampling site for ridley sea turtles (*Lepidochelys* spp.). Red circled lines depict non-rookery populations (i.e., foraging grounds, bycatch, or ghost-net). Haplotypes characteristic of Atlantic olive ridley populations are shown in blue shades, Indian in aqua shades, Indian-West Pacific in red/pink shades, and East Pacific in orange shades, while Kemp’s ridley haplotypes are shown in green. Rare haplotypes (found in less than three individuals) are not included on the map. Smaller circles indicate sampling locations with only one sample. Coordinates for all populations are approximate. Literature sources and haplotype frequencies are given in Tables S1 and S2. Putative geographical barriers are shown by dotted bars

A combination of new and previously studied samples was sequenced to obtain approximately ~ 800 base pairs (bp) of mtDNA control region and genotypes for 15 nuclear microsatellites (Fig. 1, Tables S1 and S2). Newly sequenced samples were obtained from the Southern Atlantic Ocean rookeries of Brazil, Benin, Equatorial Guinea, Gabon, Ghana, Ivory Coast, and São Tomé; as well as from several oceanic foraging grounds in the Atlantic and Pacific Oceans (Table S1). Samples resequenced for longer mtDNA fragments and genotyped for microsatellites were from rookeries in Baja California Sur, Costa Rica, French Guiana, Malaysia, Mexico, Sri Lanka, and Surinam (Bowen et al. 1997; Plot et al. 2012). Australian samples were newly genotyped for microsatellites but had already been sequenced for longer mtDNA haplotypes (Jensen et al. 2013).

Samples from nesting beaches were collected from either tissue (e.g., embryos, hatchlings, nesting females) or blood (nesting females). Different sampling and extraction methods were used per the samples’ origin as described in the original papers (Bowen et al. 1997; Plot et al. 2012; Jensen et al. 2013). For all collections, tissues were preserved in a saturated salt solution or 20% DMSO in saturated NaCl. Total genomic DNA was extracted following standard phenol-chloroform protocols (Sambrook et al. 1989). For samples from Africa, excluding Guinea-Bissau which was processed as aforementioned, high molecular weight DNA was isolated from approximately 20 mg of dermic tissue by overnight digestion at 40 °C in a lysis solution containing 200 mM Tris-HCl, 500 mM KCl and 100 ng Proteinase K. The digestion mixture was incubated at 99 °C for 15 min and subsequently stored at 4 °C (Allen et al. 1998).

### Mitochondrial DNA

For all specimens sequenced in this study (Table S1), an ~ 800 bp mtDNA control region segment was amplified using primers LCM15382 and H950 (Abreu-Grobois et al. 2006). All Polymerase Chain Reactions were carried out with negative controls. Products were checked on 1% agarose gels stained with ethidium bromide, enzymatically purified, and sequenced using a MegaBace 1000 (GE Healthcare). The remaining samples from Africa (except for Guinea-Bissau which was processed as aforementioned) were sequenced using BigDye Terminator v3.1 chemistry (Applied Biosystems) according to the manufacturer’s protocol and sequenced on an Applied Biosystems 3130xl genetic analyzer. Sequences were checked and aligned using the Clustal X method in Geneious 5.1.4 (Biomatters) and manually edited when necessary.

In addition, previously published sequences for both ridley species were downloaded from GenBank (Table S1). Some of the earliest published data were limited to short segments (~ 470 bp). Here we only included longer sequences (> 700 bp) to potentially increase power to resolve population structure. In total, 283 Kemp’s ridley (Duchene et al. 2012; Frey et al. 2014; Reid et al. 2019; Frandsen et al. 2020) and 340 olive ridley sequences were obtained from published studies (Duchene et al. 2012; Plot et al. 2012; Jensen et al. 2013; Revuelta et al. 2015; González-Paredes et al. 2017; Campista León et al. 2019; Work et al. 2019; Adnyana et al. 2020; Silver-Gorges et al. 2020; Stelfox et al. 2020). Several segments from Madagascar and India (Stelfox et al. 2020) were excluded because haplotype frequencies were not available, and only publicly accessible haplotypes from Adnyana et al. (2020) were included. Sequences were aligned and truncated to the most common overlapping region, a 653 bp segment used to analyze all 1,230 individuals. For consistency across studies, the olive ridley sequences were classified following NOAA’s nomenclature. In the absence of a similar system for Kemp’s ridleys, the haplotypes were renamed based on the order of their relative frequency (Table S2).

Basic control region genetic diversity indices were calculated in Arlequin v3.5.2.2 (Excoffier and Lischer 2010) and DNAsp v6.12.03 (Rozas et al. 2003), including the number of haplotypes (Hap), nucleotide (π), and haplotype (H_d_) diversities, polymorphic sites (PS), Fu’s F_s_ (Fu 1997) and Tajima’s D (Tajima 1983). In addition, population differentiation tests of pairwise Φ_ST_ and AMOVA were done in Arlequin. Groups with less than ten samples were excluded from population-level statistical analyses but were kept in ocean-level work. Relationships between the haplotypes were estimated using a median-joining network (Bandelt et al. 1999) implemented in PopART v1.7 (Leigh and Bryant 2015).

Genetic relationships and species divergence times were estimated using the Bayesian approach in BEAST2 v2.6.3 (Bouckaert et al. 2019). All study haplotypes were included, and five loggerhead sea turtles (*Caretta caretta*) were used as the outgroup. Details on calibration points and BEAST parameters are displayed in Supplementary Text 1.

### Microsatellites

Nuclear DNA variation was assayed for 285 global *L. olivacea* samples using fifteen microsatellite loci (Table S1). For details on genotyping, see Supplementary Text 2, and for a description of primers used, see Table S3. The microsatellite data were screened for null alleles and genotyping errors (e.g., stuttering) using Micro-Checker (Van Oosterhout et al. 2004), and linkage disequilibrium between loci was estimated using Arlequin. Although Silver-Gorges et al. (2020) found three of these loci linked in populations from Costa Rica and Mexico, no consistent linkage pattern within populations was detected when analyzing the present worldwide dataset and thus all 15 loci were retained.

The following diversity indices were calculated with Arlequin: the number of alleles per locus (K), observed (*Ho*), and expected heterozygosity (*He*) under Hardy-Weinberg equilibrium, with sequential Bonferroni corrections (p = 0.05). The Polymorphic Information Content (PIC) was estimated using Cervus v3.0 (Marshall et al. 1998). Population genetic differentiation tests were carried out in Arlequin with statistical significance calculated over 10,000 permutations (α = 0.05). *G’’*_ST_, a less biased estimator than *F*_ST_ when using a limited number of populations (Meirmans and Hedrick 2011), was calculated in GenAlEx v6.5 (Peakall and Smouse 2012).

Population genetic structure was further assessed using model-based (STRUCTURE; Pritchard et al. 2000) and non-model-based (DAPC; Jombart et al. 2010) methods. The Bayesian clustering approach implemented in STRUCTURE 2.3.4 uses an MCMC procedure to estimate the posterior probability in which the data fit the hypothesis of *K* clusters [Pr (X|*K*)]. Because of differences in sample sizes between populations, the recommended priors in Wang (2017) were employed. An uncorrelated allele ancestry prior and alpha as 0.1 were used to account for the ten sampling sites. First, the number of clusters was tested by performing 20 replicates for each *K* from 1 to 15, with 1,000,000 MCMC iterations, 10% burn-in, and no prior information on sampling location. Analyses incorporating prior population information and assuming *K* = 3 (corresponding to the Indian-West Pacific, Atlantic, and East Pacific Ocean regions) were also run to identify possible migrants or individuals with an ancestor from a different cluster. In STRUCTURE, individuals with admixture proportions (Q) from 0.2 to 0.8 were considered potentially admixed, and migrants were individuals who had Q > 0.8 of a genetic component associated with populations different from their origin (Bergl and Vigilant 2007). To align the multiple outcomes generated by STRUCTURE and determine the optimal *K*, the online tool CLUMPAK (Kopelman et al. 2015) was used.

To assess the pattern of microsatellite genetic variability among individuals, Discriminant Analysis of Principal Components (DAPC), which is based on Discriminant Analysis clustering of individuals after a Principal Components Analysis (PCA), was employed. The *dapc()* function from the R package *adegenet* (Jombart and Ahmed 2011) was used, and the optimal number of clusters was predicted using the sequential *K*-means method and then the Bayesian Information Criterion (BIC) to choose the best *K* from 1 to 20. The number of principal components that explained 90% of the cumulative variance was retained. DAPC was also run with sampling areas as prior information.

Bayesian inference was employed to estimate demographic parameters and test 21 colonization models between the three ocean regions (Fig. S1) using Migrate-n v.4.4.0 (Beerli and Felsenstein 2001). Demographic parameters estimated (using an island model) were theta (as 4*Neµ*, where *Ne* is the effective population size and *µ* is the neutral mutation rate per site per generation) for each ocean region, and the asymmetrical immigration rate (M = m/*µ*) between each one. The best colonization model was estimated using their marginal likelihoods. Details of the method are in Supplementary Text 1.

Finally, an Approximate Bayesian Computation approach (ABC) implemented in DIYABC 2.1.0 (Cornuet et al. 2014) was used to infer olive ridley demographic history. For each oceanic region, three models (stable population, population reduction, and population expansion, Fig. S2) were tested. Prior distributions for the mutational model used are in Fig. S3, and priors for the demographic parameters for each model are in Table S4. All available one-sample summary statistics in DIYABC were used. One million datasets were simulated for each scenario, and their posterior probabilities were assessed through logistic regression using the best 10,000 (1%) simulations. For each best scenario, the posterior distribution of recent (*Ne*) and past (*Na*) effective sizes and the time of size change (t, in thousands of years) were estimated using logit transformation for the 10,000 best simulations.

## Results

### mtDNA diversity and phylogeography

The 653 bp-long mtDNA alignment was obtained for a total of 1,230 ridley sequences (n_*L. kempii*_ = 287; n_*L. olivacea*_ = 943) and included 558 new unpublished sequences for both species (n_*L. kempii*_ = 4). Nine Kemp’s ridley haplotypes with nine substitutions were detected (Tables S1 and S2). There were 53 olive ridley haplotypes containing 49 polymorphic sites, and 732 samples came from *L. olivacea* rookeries. The number of olive ridley haplotypes and haplotype diversity varied between the three ocean regions (Table S1). The lowest number of haplotypes was found in the Atlantic (ATL, n = 8), followed by the Eastern Pacific (EP, n = 16) and the Indian-West Pacific (IWP, n = 17).

**Fig. 2:**
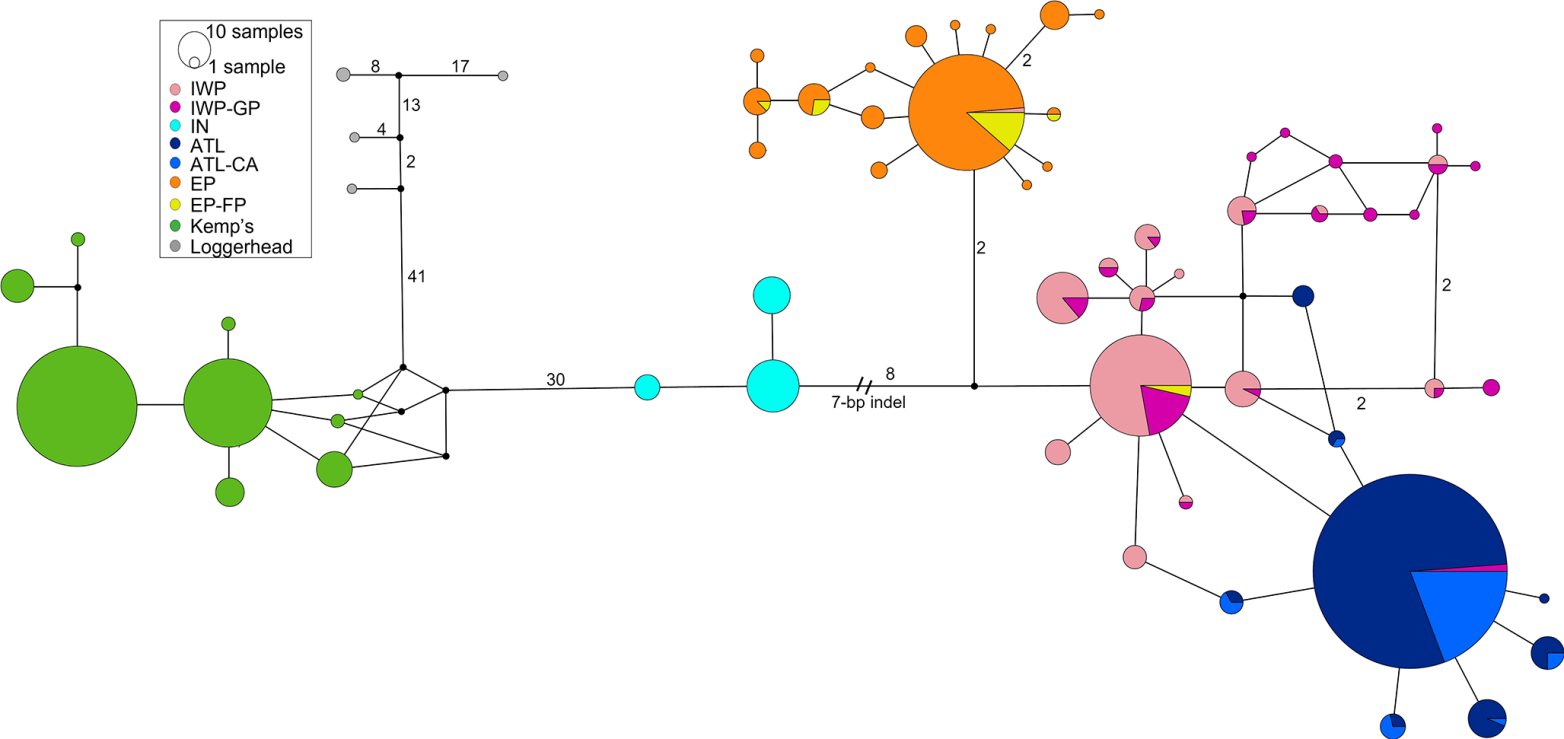
Median-joining network showing the relationships among Kemp’s and olive ridley mtDNA 653 bp control region haplotypes. Circles are proportional to the number of individuals. Black circles indicate missing haplotypes, and two or more mutational steps are shown as numbers. Colors represent sampled regional rookeries and non-rookeries as described in Fig. 1. Loggerhead sea turtles (the outgroup) are shown in gray. IWP = Indian-West Pacific rookeries, IWP-GP = Indian-West Pacific Ghost net and Pelagic, IN = Indian nesting and foraging, ATL = Atlantic, ATL-CA = Atlantic feeding and Capture at-sea, EP = East-Pacific rookeries, EP-FP = East Pacific Foraging and pelagic/stranded

The mtDNA haplotype network (Fig. 2) revealed that Kemp’s and olive ridley haplotypes are highly divergent from each other and separated by 30 mutational steps. However, relatively few substitutions separated olive ridley regional clades. The most divergent haplotype group, distinguished from the other haplotypes by at least nine substitutions, was found in the IWP region comprising India, the Maldives, Oman, Sri Lanka, and West Australia (Fig. 2). This divergent haplotype group corresponds to the previously reported “clade K” (haplotypes Lo44 and Lo47-51 in the standardized nomenclature), which had been described with shorter sequences (~ 400 bp) from Sri Lanka (Bowen et al. 1997) and India (Shanker et al. 2004). Furthermore, as shown in Fig. 2, this divergent haplotype group has a seven bp indel shared with four other sea turtle species (loggerhead, hawksbill, leatherback, and green sea turtles; Shanker et al. 2004) but not present in other olive ridley clades. One or two substitutions separated the regional clades, while the most frequent haplotypes within each ocean region presented a star-like pattern with a central sequence from which others branched off. The most common ATL haplotype (Lo67) was separated from the IWP clade by one substitution (Fig. 2). Geographic structure between ocean regions was indicated as only one breeding female with an mtDNA haplotype from another region was found: a female nesting in eastern Australia carried a typical Eastern Pacific haplotype (Lo27). However, in the bycatch and ghost net samples, there was evidence of unidirectional movements from EP to IWP (Korea) and from ATL to IWP (Madagascar) (Figs. 1 and 2, Table S2).

Pairwise Φ_ST_ comparisons between rookeries within ocean regions showed that the lowest Φ_ST_ measures were between populations within the Atlantic and within the Eastern Pacific (Φ_ST_ = 0 to 0.15) (Table S5). Within the Indian-Western Pacific there were significant Φ_ST_ values, suggesting strong intra-regional population structure. Conversely, there was no difference between Oman and Sri Lanka (~ 3,000 km distance), the two rookeries that bore haplotypes from the endemic and basal Indian Ocean clade.

AMOVA analysis was run using groupings formed based on rookery pairwise Φ_ST_ comparisons, the Median-Joining Network, and haplotypic geographic distribution patterns (Figs. 1 and 2). Olive ridley sequences were grouped into four ocean regions (Indian, Indian-West-Pacific, Eastern Pacific, and Atlantic). AMOVA results confirmed that olive ridley rookeries are structured between these four ocean regions with Φ_ST_ = 0.69 (p < 0.001). Between regions, pairwise Φ_ST_ values were also high (ATL x IWP = 0.61, ATL x EP = 0.87, IWP x EP = 0.66, IN x ATL = 0.80, IN x IWP = 0.52, IN x EP = 0.67; p < 0.001 for all comparisons).

**Fig. 3:**
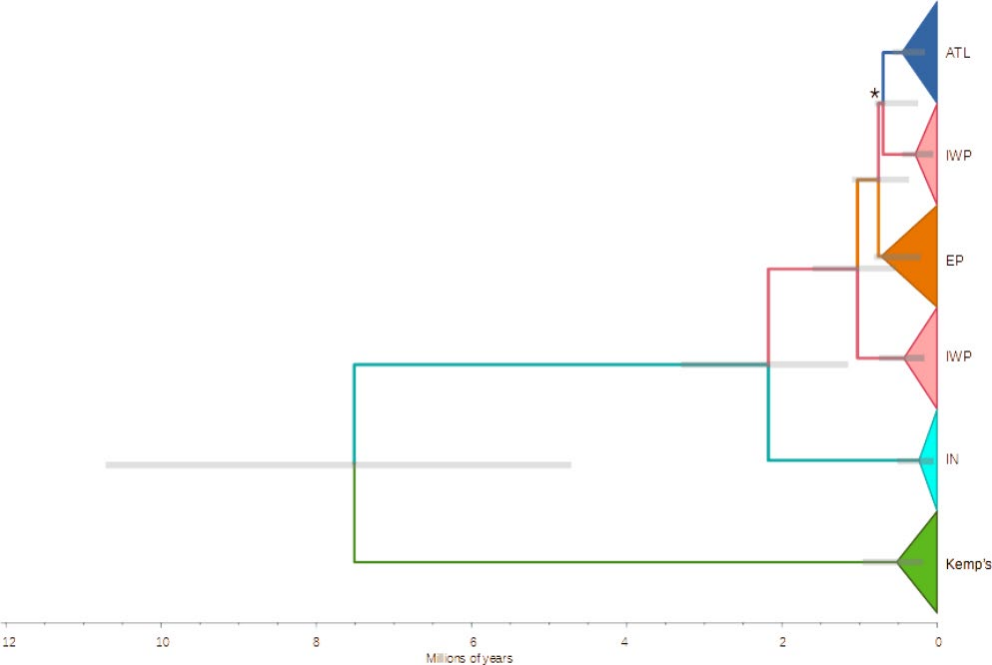
Bayesian tree with divergence times of olive ridley mtDNA sequences (ATL, IWP, EP, IN) and Kemp’s ridleys. Abbreviations and colors as in Fig. 2. Terminal clades were represented by triangles in which deep points correspond to diversification times. Loggerhead sea turtles were used as the outgroup (not shown). Gray bars represent 95% High Posterior Density estimates for divergence times. The asterisk denotes the only major clade subdivision with low Posterior Probability values (PP = 0.02)

The Bayesian phylogenetic tree (Fig. 3) was concordant with the mtDNA haplotype network (Fig. 2) and revealed three divergent and well-supported clades: (1) Kemp’s ridley; (2) Indian Ocean olive ridley; (3) all other olive ridley haplotypes. This last group is further divided into one Atlantic (ATL), one East Pacific (EP), and two Indian-West Pacific (IWP) subclades. The posterior probability (PP) of all nodes was high (PP > 0.88), except for the ATL and one IWP subclade (PP = 0.02). The split between the two ridley species was estimated at 7.5 Mya (95% High Posterior Density (HPD) = 4.7–10.7 Mya). The basal clade in the Indian Ocean (IN) was estimated to have split 2.2 Mya (95% HPD = 1.1–3.3 Mya), while the ATL/EP/IWP clade diverged 1.0 Mya (95% HPD = 0.5–1.6 Mya). Diversification times within these groups were all recent (< 520,000 YBP).

### Olive ridley microsatellite population structure and demography

In total 285 individuals from 12 globally distributed olive ridley sites, of which 11 were rookeries and one was a feeding ground, were genotyped (Table S1). Almost all loci were polymorphic in the sampled populations. On average, 95% of the individuals were genotyped entirely, but a dataset of 227 individuals (including 11 from the EP feeding ground) that presented at most one missing marker was used for DAPC and STRUCTURE analyses. Similar to the mtDNA results, Atlantic olive ridley populations had lower diversity values than those of the East-Pacific and Indian-West Pacific (Table S1).

**Fig. 4:**
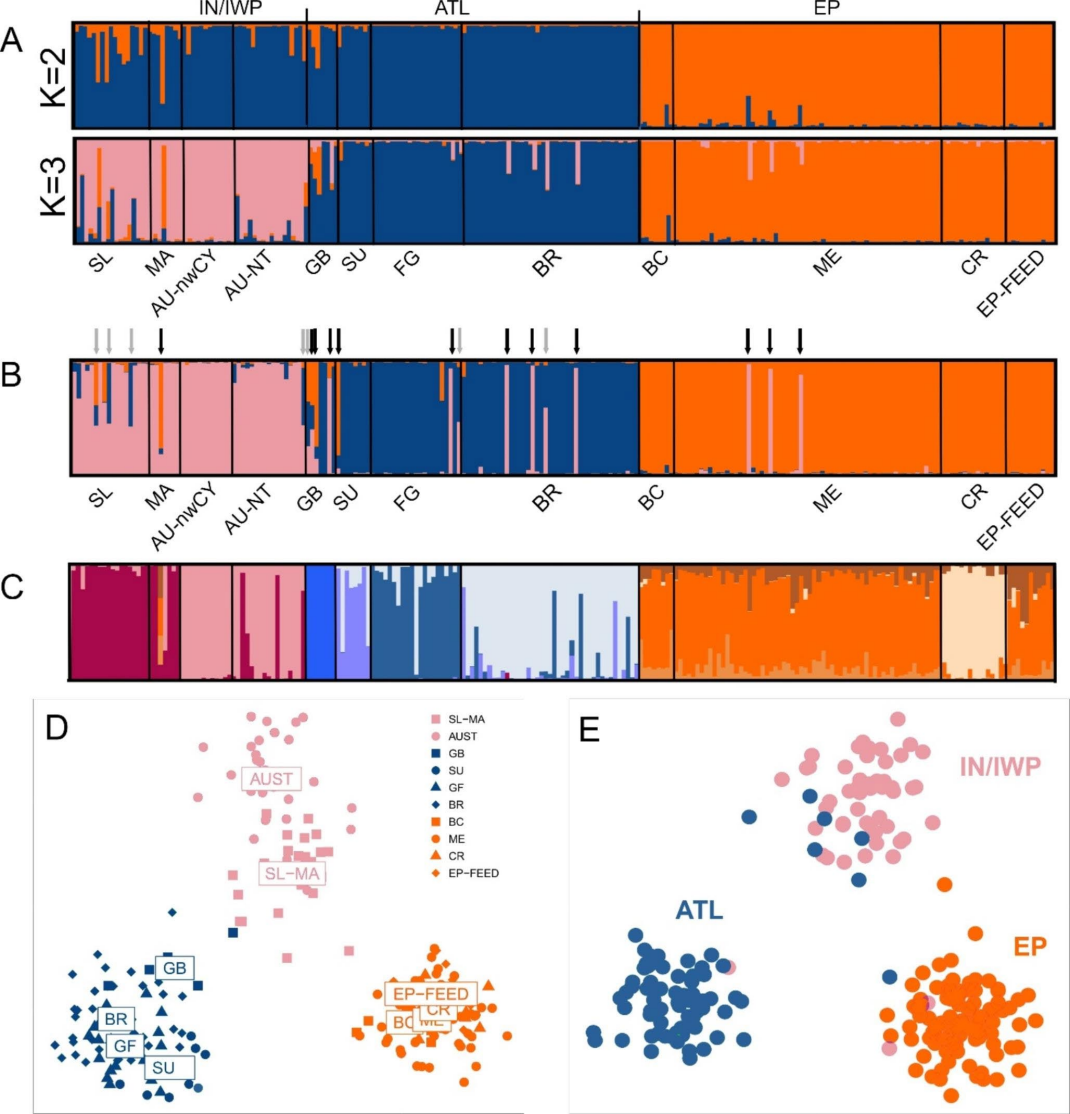
(A) STRUCTURE barplot of individual admixture proportions from olive ridley microsatellite genotypes for *K* = 2 and *K* = 3 without prior population information. (B) STRUCTURE barplot using oceanic region of origin as prior information. Arrows show individuals with *Q*-values from 0.2 to 0.8 (admixed, grey arrows) or recent migrants (Q > 0.8, black arrows). (C) DAPC barplot with membership probabilities, similar to the STRUCTURE barplots, with *K* = 12 and the 12 main sampling sites (in the same order as in A-B) as prior information. (D) DAPC scatterplot with *K* = 3 and the 12 main sampling sites as prior information. (E) DAPC scatterplot with *K* = 3 without prior population information. Codes used below the horizontal axis represent sample collection location. Only individuals with a maximum of one missing locus are shown. SL = Sri Lanka; MA = Malaysia; AU-nwCY = Australia north-western Cape York; AU-NT = Australia Northern Territory; GB = Guinea-Bissau; SU = Surinam; FG = French Guiana; BR = Brazil; BC = Baja California; ME = Mexico; CR = Costa Rica and EP-FEED = East Pacific (Mexican waters) foraging grounds

The olive ridley STRUCTURE analysis showed *K* = 3 or *K* = 2 with the highest likelihood depending on the method (Fig. S4). Nonetheless, there was clear geographic differentiation between ocean regions (Fig. 4 A). Runs using default and location priors showed similar grouping results. At *K* = 2, the separation was between IN/IWP/ATL x EP, indicating higher similarity between the Atlantic and Indian-West Pacific. At *K* = 3, there was clear geographical separation corresponding to the three major ocean regions (ATL, EP, and IN/IWP). Unlike the mtDNA pattern, a distinctive Indian Ocean group was not found using nuclear microsatellites. Running similar STRUCTURE analyses for each major ocean region separately revealed no finer-scale population differentiation, indicating prevalent dispersal within ocean regions (Figs. S5 and S6). Migration analysis with STRUCTURE using ocean regions as location priors (K = 3) revealed that 11 of the 216 individuals sampled from the rookeries were likely migrants, and an additional seven appeared to be admixed between ocean regions (Fig. 4B). The movement was asymmetrical: while EP and ATL received individuals from IN/IWP, ATL and IN/IWP received only one individual each from EP. Within the Atlantic, Guinea-Bissau (n = 8) stood out as mainly composed of migrants (n = 3) and admixed individuals (n = 3) from both EP and IN/IWP. Sri Lanka and Malaysia had admixed turtles, but neither Australian population had migrants or admixed individuals. The only feeding area genotyped here, from the Eastern Pacific, consisted exclusively of turtles of EP origin.

The DAPC results were similar to those of STRUCTURE. Most areas presented distinct genetic components in comparison to each other, but were relatively homogeneous within, except for the EP in which only Costa Rica was differentiated (Fig. 4 C). Analysis with the 12 sampling locations given as prior groupings highlighted the main divisions between the three ocean regions (ATL, EP, and IN/IWP), with further site separations within ocean regions (Fig. 4D). Individuals from the EP feeding area in Mexican waters mostly displayed a genetic profile characteristic of Mexico, and not Costa Rica. In the DAPC based on individuals’ prior grouping into the three ocean regions (ATL, EP, IN/IWP), all turtles were correctly classified to their area of origin (Fig. S7). The analyses without prior groupings indicated that four was the best number of clusters, although *K* = 3 was very close (Fig. S8). The three clusters in the *K* = 3 analysis without prior grouping mostly matched the main ocean areas (Fig. 4E), but possible migrants were detected mainly from IN/IWP to ATL, as also revealed in the STRUCTURE results. At *K* = 4, EP and IN/IWP formed single clusters, while ATL split into two groups with no geographical or other apparent subdivision (Fig. S9).

Similarly, pairwise *F*_ST_ and *G*’’_ST_ estimates between rookeries showed that the genetic composition of some populations within the same ocean region was not significantly different (Tables S6 and S7). Guinea-Bissau had the highest divergence values among Atlantic Ocean rookeries, indicating structure between the eastern and western Atlantic. Differentiation values were low within the East Pacific, although statistically significant between Mexico and Costa Rica. Indian-West Pacific populations also had low *F*_ST_. However, *F*_ST_ values between populations outside these three oceanic regions were significantly different and usually higher than within regions.

As also found with STRUCTURE and DAPC, Migrate-n migration rate estimates revealed high gene flow between IN/IWP and ATL, although in this case, in the opposite direction, from ATL to IN/IWP. Significantly more individuals per generation (ipg) were detected immigrating to IN/IWP from ATL (median 100 ipg) than to IN/IWP from EP (median 15 ipg). This rate was higher than to ATL from both EP and IN/IWP (median ~ 4 ipg) (Tables S1 and S8). Moreover, the historical *Ne* estimates for the three ocean regions were significantly different, with median values estimated at 1,302 for the Atlantic, 2,208 for the East Pacific, and 1,769 for the Indian-West-Pacific (Table 1 and S8). However, in contrast to the above results, the Migrate-n best divergence/migration scenario showed a closer relationship between EP and ATL, indicating step-wise colonization from IN/IWP →EP →ATL (Model probability = 1) (Fig. S1).

**Table 1:** *Ne* (diagonal) and the number of immigrants per generation estimated using Migrate-n for the three oceanic regions with µ = 5.7E-4. Values are shown as median followed by 95% confidence interval (in squared brackets). IN/IWP = Indian /Indian-West Pacific, ATL = Atlantic, EP = East-Pacific

	IN/IWP	ATL	EP
IN/IWP ->	1,769 [1,404-2,018]	3 [1–6]	9 [4–12]
ATL ->	151 [121–202]	1,302 [1,053−1,433]	11 [6–14]
EP ->	15 [10–19]	4 [1–5]	2,208 [2,106-2,340]

ABC model selection analysis showed that the population expansion model had the highest posterior probability in all three ocean regions (Fig. S2). The ancestral population size (*Na*) was similar for all three areas, while recent estimates showed EP to have the largest *Ne*, followed by ATL and IN/IWP (Table 2). Although their confidence intervals overlap, the expansion time point estimates for each major population differed; IN/IWP and EP expansion occurred before the LGM (~ 50,000 and ~ 30,000 YBP, respectively), while ATL expansion followed the last glacial maximum (~ 10,000 YBP) (Table 2).

**Table 2:** Posterior estimates (mode and 95% confidence interval within parenthesis) of recent (*Ne*) and past (*Na*) effective population size (in thousands). Time (*t*, in thousands of years) of size changes for each major ocean basin population for the expansion scenario is displayed. See Fig. S2 for details of the scenario and Table S4 for the priors. Abbreviations as in Table 1

Population / Parameter	IN/IWP	EP	ATL
*Ne*3	50.7 (15–195)	485 (26–489)	153 (9.7–196)
*t*	48.4 (8.8–97)	28 (6.2–57.6)	9.8 (3.2–38.4)
*Na*3	1.0 (0.2–4.7)	0.8 (0.2–4.5)	0.8 (0.3–4.5)

## Discussion

Here we report, to our knowledge, the first global study of a sea turtle genus using both mitochondrial and nuclear markers. While other works have used similar molecular data, they were all limited to a single ocean basin or region (Fitzsimmons et al. 1997b; Dutton et al. 1999; Bowen et al. 2005; Carreras et al. 2007; Monzón-Argüello et al. 2010) or the species had a restricted geographical distribution (FitzSimmons et al. 2020). Here, we describe global connectivity patterns, differential sex-dispersal, genetic diversity, and / or biogeography with conservation applications in threatened *Lepidochelys*.

### Ridley historical biogeography

The ~ 7.5 Mya point estimate for ridley speciation derived from our study’s robust mitochondrial sequence data set falls well before the final closure of the Panama Isthmus (~ 2.8Mya, O’Dea et al. 2016), and earlier than previously hypothesized (Bowen et al. 1991; Naro-Maciel et al. 2008; Duchene et al. 2012). This estimate coincides with that of Pacific and Atlantic green sea turtle divergence, in which gene flow between the Indo-Pacific and Atlantic Oceans was thought to have been disrupted by the Tethys Sea closure and late Miocene southern ocean cooling (Naro-Maciel et al. 2008). We additionally hypothesize that, following the changes in ocean currents, temperatures, and salinity, the onset of glaciation in the Northern Hemisphere 3.1–2.5 Mya further isolated Kemp’s ridleys in the Gulf of Mexico. However, caution is warranted when interpreting this result because its lower confidence interval limit overlaps with prior estimates of Isthmus-related speciation, and this part of the study was based solely on the mtDNA control region.

Our results agree with previous findings that the mitochondrial IN clade (clade “K”), endemic to the Indian Ocean and dated herein to ~ 2.2 Mya, is the most divergent lineage of olive ridley haplotypes (Bowen et al. 1997; Shanker et al. 2004). The persistence of this clade suggests long-term olive ridley survival in the Indian Ocean, where populations may have been protected from Isthmus-related disruptions by distance, and also warmed by the ancient Indonesian Seaway 3–5 Mya (Cane and Molnar 2001). The climatic stability and sea surface temperature of the Indian Ocean before and throughout the Pleistocene contrasts with the Isthmus-derived unstable conditions in the Eastern Pacific and Northern Atlantic (Lambeck et al. 2002; Nishimura 2002), which caused significant reorganization of ocean circulation and other effects (Haug and Tiedemann 1998).

The remaining olive ridley clades coalesce much more recently (~ 1 Mya) than the split of IN lineage, as expected given the climatic instability of other oceanic areas. Previous work hypothesized recent East Pacific and Atlantic (re)colonization from the Indian Ocean (Shanker et al. 2004). Our mtDNA and microsatellite results are consistent with this scenario, but we argue that biogeographical inferences based solely on mtDNA must be approached carefully given the limitations of relying on a single genetic marker. A history of recurrent extinction and recolonization would be expected to result in recent and shallow divergence patterns without requiring recolonization from a single source (Avise 2000), such as the Indian Ocean. Therefore, extant mtDNA phylogeographic patterns likely do not represent the original history of olive ridley colonization of ocean regions, but only the most recent realization of a pattern of recurrent extinctions and recolonizations.

The hypothesis of recent (< 1 Mya) recolonization of the Atlantic from the Indian-West Pacific is upheld by the sister relationship between ATL and IWP clades (Fig. 3) in addition to the IWP-derived central mtDNA haplotype in the ATL (Fig. 2). This close link between Atlantic and Indo-West Pacific populations is also supported by most microsatellite results (Fig. 4 A). In contrast, extant EP and ATL lineages are not directly related (Fig. 2) and coalesce much more recently (~ 1 Mya) than expected if the Atlantic Ocean had been colonized from the East Pacific via the Indo-Pacific after the Panama closure (Pritchard 1969). The Migrate-n best divergence model for microsatellites (that ATL was colonized from EP) conflicts with our other results indicates a complex demographic history likely requiring population genomic investigation.

In comparison to other sea turtles, olive ridley populations have shallow evolutionary histories (Naro-Maciel et al. 2008; Duchene et al. 2012) with mtDNA haplotypes within each clade coalescing fairly recently (300,000–600,000 YBP). The extended mtDNA network, characterized by star-shaped trees with few mutational steps in each major clade (Fig. 2), is compatible with synchronous post-LGM (~ 10,000 YBP) expansions reported for all major ridley and other sea turtle lineages (Reid et al. 2019). Here for the first time, we provide evidence of recent population size expansions from nuclear microsatellites around the LGM (Table 2 and S1).

### Biogeographic barriers to dispersal

The Cape of Good Hope in southern Africa is considered a barrier between the Indian and Atlantic Oceans for some tropical organisms (Avise 2000; Bowen and Karl 2007). However, the olive ridley haplotype typical of the Atlantic (Lo67) was found in Madagascar bycatch indicates some linkages between ATL and IWP, even if restricted to the east African coast. Moreover, microsatellite data detected several migrants between these regions. Indeed, marine turtle migrations between the Atlantic and Indian Oceans have been previously reported, including for green (Bourjea et al. 2007), hawksbill (Vilaça et al. 2013), and loggerhead turtles (Shamblin et al. 2014). Typical Atlantic green turtle haplotypes such as CM-08 were found at several southwest Indian Ocean nesting sites (Bourjea et al. 2007). Regarding hawksbills, Vilaça et al. (2013) described a sequence from Iran (EiBR-14) displayed at a northeast Brazilian feeding area. A loggerhead haplotype typical of South Africa (CC-A2) was found in the Brazilian Rio Grande Rise foraging aggregation along with loggerhead haplotypes common in Australia (CC-A33 and CC-A34) and Oman (CC-A11), further revealing Atlantic and Indian-West Pacific linkages (Shamblin et al. 2014). Although limited to one haplotype, our findings combined with evidence from other studies do not support the hypothesis that the Cape of Good Hope is an impermeable barrier to sea turtle migration.

In contrast, due to frigid temperatures and inhospitable conditions, Cape Horn at the tip of South America represents a considerable biogeographic barrier to ridley dispersal, with no sign of connectivity found between East Pacific and Atlantic (Figs. 1 and 4). Within the Pacific Ocean, however, we found evidence of both mtDNA (Fig. 1) and microsatellite (Table 1; Fig. 4B) gene flow between EP and IWP, indicating that olive ridleys are capable of long-distance migrations. Transpacific movements between rookeries and foraging aggregations have been reported in loggerhead and green turtles (Nichols et al. 2000; Nishizawa et al. 2014). Satellite telemetry revealed that although some olive ridleys are nomadic, others occupy neritic waters or undertake extensive migrations depending on the ocean of origin (Plotkin 2010). These patterns support our results in that broad conclusions regarding the migratory behavior of olive ridleys should not be drawn due to variation between oceans/populations. While this may complicate conservation strategies (Rees et al. 2012), migration between oceans might also facilitate the recolonization of depleted rookeries, and from an evolutionary standpoint, it could be beneficial to have occasional gene flow across different ocean basins.

### Population structure

The highly significant olive ridley mtDNA structure between ocean regions (Figs. 1 and 2, Table S5) corroborates previous results from a limited dataset of 80 shorter sequences, and suggests high female philopatry at this level (Bowen et al. 1997). In contrast to findings in loggerheads (Shamblin et al. 2014) and hawksbills (Leroux et al. 2012), the longer mtDNA control region sequences (~ 653 vs. ~400 bp) did not provide substantially higher resolution in detecting new population structure; instead, the results were similar to those reported previously (Bowen et al. 1997; Shanker et al. 2004). We found that only one of the 732 olive ridley mtDNA rookery sequences corresponded to haplotypes from a different ocean region (an EP haplotype found in Australia), showing that female single-generation dispersal between ocean regions is rare, a pattern observed in most sea turtles (Reid et al. 2019). Here, for the first time, this pattern of significant population structure between ocean regions is corroborated with nuclear genetic (microsatellite) data (Fig. 4).

However, the near absence of mtDNA (female-mediated) movement between these areas (Fig. 1) does not preclude more complex scenarios of some male-facilitated gene flow (e.g., at regionally-shared feeding or breeding habitats or along migratory routes; FitzSimmons et al. 1997a). Indeed, we found more instances of individuals with mtDNA markers from a different ocean region in mixed-sex inwater samples, although still in small numbers (n = 7). In addition, six olive ridleys of Eastern Pacific origin and three from the Western Pacific were detected in the northern Central Pacific (Polovina et al. 2004), indicating sharing of pelagic foraging habitats. Furthermore, our migration analyses using microsatellite data supported recent (migrant individuals) and past (mixed ancestry samples) migration between oceanic regions, particularly ATL and IWP (Fig. 4B and E; Table 1). Therefore, our nuclear and mitochondrial results corroborate recent but low gene flow between ocean regions and suggest that either males are the main drivers of dispersal, or that mating occurs when movements overlap.

Within ocean regions, olive ridley mtDNA differentiation among regional rookeries ranged from moderate to non-significant, and populations were less structured than in other sea turtle species. This pattern could indicate relatively recent colonization, as suggested above, or generally low levels of natal philopatry. In favor of the latter hypothesis, in Costa Rica some solitary nesting females breed at multiple beaches hundreds of kilometers apart during the same nesting season (Morreale et al. 2007). In contrast, olive ridley females from Australian rookeries swim up to 40 km away but return to re-nest on the same beach (McMahon et al. 2007; Whiting et al. 2007; Hamel et al. 2008). Similarly, tagging data from Brazil suggest that olive ridley nesters are faithful to their rookeries (Matos et al. 2012). No females tagged there have been reported nesting in neighboring Surinam or French Guiana (~ 3,000 km distance) (Da Silva et al. 2007), although their post-nesting behavior includes movements towards foraging areas in French Guiana and West Africa (Santos et al. 2019).

In our analysis, the more likely explanation for this low mtDNA differentiation is the relatively recent origin (by recolonization) of most *L. olivacea* nesting areas. Even if isolated by philopatric behavior, structure between most of these rookeries would be shallow following relatively recent recolonization after local extinctions. Given the mtDNA mutation rate (see Reid et al. 2019), the time necessary to establish even small (2–3 mutations) sequence differences between clades is tens to hundreds of thousands of years. The recent origin explanation was further corroborated by analyzing more rapidly evolving microsatellites, in which population differentiation between some rookeries within oceanic regions was significant (Fig. 4 C, Table S6).

The DAPC results with sample sites as priors indicated that all nesting areas, except Mexico and Baja California, presented characteristic genetic profiles despite some inter-rookery migration within oceanic regions (ATL, EP, IN/IWP; Fig. 4 C). While Mexico and Baja California do not display significant population structure, our study only had eight samples from Baja California. Therefore, we cannot discard the possibility that enhanced sampling might detect differentiation between these two populations. Overall, our microsatellite results suggest that olive ridleys maintain low but significant biparental (nuclear) genetic differentiation between rookeries in the same ocean region, indicating that nesting areas within oceans have greater genetic structure than previously estimated.

Finally, stronger population genetic structure has repeatedly been reported in mitochondrial than nuclear markers (loggerheads: Bowen et al. 2005; Carreras et al. 2007; Monzón-Argüello et al. 2010), (green turtles: Fitzsimmons et al. 1997b; Naro-Maciel et al. 2014), (leatherbacks: Dutton et al. 2013; Molfetti et al. 2013), (flatbacks: FitzSimmons et al. 2020), (hawksbills: Vilaça et al. 2012, 2013). The higher mtDNA population structure can be attributed to mitochondrial *Ne* being four times lower than its nuclear counterpart, which accelerates drift amid characteristic female philopatric behavior. The differences between mtDNA and nuclear markers are possibly also attributed to either male-mediated dispersal from diverse regional rookeries (i.e., lower male philopatry) (Bowen et al. 2005) or opportunistic mating during overlapping migrations (Fitzsimmons et al. 1997a). In this latter case, philopatric males and females from different stocks might breed in foraging areas or migratory routes despite remaining faithful to their natal breeding grounds. Our results suggest that in the olive ridley, the shallow mtDNA structure is likely a consequence of relatively recent population differentiation. However, it remains to be determined whether ridley males are equally as or less philopatric than females at the regional level, and if mating happens during overlapping migrations as suggested for some green turtles in Australia (Fitzsimmons et al. 1997a).

### Genetic diversity

Consistent with their recent evolutionary history, ridley species have lower genetic diversity than other globally-distributed sea turtles. The Atlantic olive ridley displayed the lowest genetic diversity of the three ocean regions for both microsatellites and mtDNA, the latter having only eight haplotypes in 363 rookery individuals, all differing by a single substitution (Fig. 2, Tables S1 and S2). This level of mtDNA haplotype divergence is comparable to Atlantic leatherbacks (Dutton et al. 2013), while other sea turtle species have higher genetic diversity within the Atlantic (Shamblin et al. 2014; Jensen et al. 2019; Arantes et al. 2020). In the Indo-Pacific, olive ridleys display more pronounced genetic diversity than in the ATL, similar to green sea turtles (Jensen et al. 2019) and hawksbills (Vargas et al. 2016; Arantes et al. 2020). However, ridleys have the unique divergent mtDNA clade in the Indian Ocean.

Consistent with their distinct species status, Kemp’s ridleys displayed haplotypes that were unique and divergent from olive ridleys. Kemp’s also had low genetic diversity comparable to a single olive ridley ocean region (Table S1). In comparison to flatbacks (*Natator depressus*, the other endemic sea turtle species) (FitzSimmons et al. 2020), Kemp’s ridleys have fewer haplotypes (9 vs. 32), and lower haplotype diversity (0.60 vs. 0.76) and mean pairwise distance (0.86 vs. 1.05). However, our study has fewer samples for Kemp’s than were available for flatbacks (287 vs. 784) (FitzSimmons et al. 2020), and other less frequent haplotypes might still be uncovered with more exhaustive sampling.

Although olive ridleys are the most abundant sea turtle species (Abreu-Grobois and Plotkin 2008; Shanker et al. 2021), they display relatively low genetic diversity. As discussed above, olive ridleys display mass nesting behavior (i.e., arribadas) that drives local numbers of individuals. Even though we did not assess the genetic diversity of specific arribadas, regions containing this phenomenon (e.g., Costa Rica and India) were not characterized by higher genetic diversity than other global populations (Table S1). This pattern was also reported in previous arribada populations that did not show significantly more alleles. Along India’s east coast, where arribadas of ~ 100,000 nesting females occur, eight haplotypes, mainly from the basal IN clade, were found (Shanker et al. 2004, 2021). A similar pattern was revealed herein for Mexico and Costa Rica, where even bigger arribadas occur (Table S1) (Shanker et al. 2021). We detected more haplotypes (12 and 9, respectively) in these populations and slightly higher mtDNA diversity statistics but similar microsatellite diversity (Table S1). However, the mtDNA diversity was not as high as expected in comparison to populations with 100 − 10,000 times fewer females/year. Therefore, we recommend that future research focus on understanding the interplay between the massive presence of ridleys and low genetic diversity from an evolutionary genetics perspective. Furthermore, it is crucial to continue monitoring ridley populations while encouraging global collaborations to ensure that population declines are not increasing inbreeding and potentially decreasing individual fitness within oceanic regions.

### Conservation genetics

The study provides key insights into conservation genetics and management strategies for both species. Given the low genetic diversity of the Critically Endangered Kemp’s ridleys (comparable to one olive ridley ocean basin) and their limited nesting range in the Gulf of Mexico, particular conservation efforts should be implemented to ensure no further population declines that could affect their long-term survival.

The more widespread olive ridleys are classified into eight Regional Management Units (RMUs): two in the Atlantic Ocean (east and west), three in the Indian (northeast, northeast arribadas, and west), and three in the Pacific (east, east arribadas, and west) (Wallace et al. 2010). Overall, our results are broadly consistent with these delineations. The Indian Ocean hosts nesting sites with divergent *L. olivacea* lineages (Shanker et al. 2004) and represents a critical conservation region due to its evolutionary significance and the occurrence of arribadas. However, we emphasize that nesting aggregations within IN/IWP and ATL had distinct genetic identities at a more refined scale than encompassed by the current RMU system (as also shown by Madduppa et al. (2021) for IWP). The present study suggests that olive ridleys can display finer-scale structure within ocean regions and that several RMUs might be more appropriate for the Indo-Pacific and Atlantic. Our data show that within IN/IWP, Australia has two Management Units (MUs), while Malaysia and Sri Lanka represent a single MU.

Within the Atlantic, microsatellite *G’’*_ST_ estimates showed that all populations might constitute a single MU, albeit with some differentiation between east and west Atlantic. This distinction is not as clear for mtDNA because one frequent haplotype is present in all populations (Lo67) and many haplotypes diverge by a single base pair from this frequent sequence (Fig. 1; Tables S1, S5). However, the distinct and possibly private haplotypes in each of the Atlantic populations analyzed are indicative of finer structure not captured in our study and warrant further investigation. Moreover, detailed research of Southeast Asian and African coastal populations where multiple solitary nesting sites are present but not fully mapped, is needed to assess the connectivity and genetic diversity of rookeries. In addition, several small, declining rookeries within each region need specific protection to halt declines or avoid population extinctions, and genetic monitoring can help estimate relationships with nearby areas and reveal unique components of genetic diversity. Long-term genetic monitoring of arribada and solitary nesting sites will help elucidate how these two behaviors contribute to maintaining genetic diversity and gene flow between rookeries.

Although population structure, gene flow, and diversity are included in all RMU assessments (Wallace et al. 2010), estimates of heterozygosity coupled with population-level *Ne* patterns are not. Effective population size is a crucial estimator of extinction risk as it influences the loss of genetic diversity and maintenance of evolutionary potential (Garner et al. 2020). Therefore, estimates of (historical and recent) *Ne* are fundamental to better assess genetic erosion and improve the IUCN Red List and RMU criteria (Garner et al. 2020). Our effective population size estimates for each ocean region yielded small current *Ne* values for the ATL and IN/IWP. Olive ridleys are the most abundant sea turtle species globally (Abreu-Grobois and Plotkin 2008; Shanker et al. 2021), but their genetic *Ne* is much lower than the census size (*Nc*) (Table S1), in agreement with our finding of very recent regional population expansion (Fig. S2). For instance, our parameters for the Atlantic Ocean are broadly concordant with recent *Ne* estimates of ~ 1,000 for Brazilian olive ridleys based on whole genomes (Vilaça et al. 2021) and mtDNA (Reid et al. 2019) (Table 1). The small *Ne* we found in the IN/IWP is likely the result of population reductions and generally low long-term genetic diversity (Vilaça et al. 2021) coupled with the extinction/colonization model supported here.

There is no doubt that human exploitation of olive and Kemp’s ridleys and other current threats have severely reduced some populations (Cornelius et al. 2007; Stelfox et al. 2019). For example, the Surinam olive ridley rookery declined from 2,800 nests in the 1960s to around 100–150 in the early 2000s (Hilterman et al. 2008; Kelle et al. 2009), and olive ridleys from the Maldives were substantially impacted by ghost nets (Stelfox et al. 2019). Similar problems persist worldwide, although conservation efforts highlight that decades-long programs can successfully reverse declining trends at regional scales (Marcovaldi and Dei Marcovaldi 1999; Da Silva et al. 2007). However, it remains to be determined how much these declines decreased population genetic diversity in these long-lived species with protracted generation times (Abreu-Grobois and Plotkin 2008; Vilaça et al. 2021). Although most anthropomorphic population reductions may be too recent to detect with most genetic tools, extensive sampling with dense genome-wide markers may be efficient for detecting the effects of very recent decreases in *Ne*. As ridleys are known for their oceanic habit, distant migrations, and long generation times, conservation and management should be carried out by international agreements and cooperation considering all life stages and migratory routes used by the species in the different oceans.

## Electronic supplementary material

Below is the link to the electronic supplementary material.

Supplementary Material 1

Supplementary Material 2

Supplementary Material 3

## Data Availability

All sequences and their frequencies are available in Table S1. Microsatellite data will be made available upon acceptance.
