# Supplementary material for: G﻿lobal phylogeography of ridley sea turtles (Lepidochelys spp.): evolution, demography, connectivity, and conservation
Source: Conserv Genet. 2022 Aug 29;23(6):995–1010. doi: 10.1007/s10592-022-01465-3 (PMC9659502; doi:10.1007/s10592-022-01465-3)
Supplement: Supplementary file 1 — Supplementary Material 1 [file 10592_2022_1465_MOESM1_ESM.docx]

**Global phylogeography of ridley sea turtles (*Lepidochelys* spp.): Evolution, demography, connectivity, and conservation**

Sibelle Torres Vilaça^1†^, Anelise Torres Hahn^2†^, Eugenia Naro-Maciel^3^, F. Alberto Abreu-Grobois^4^, Brian W. Bowen^5^, Jaqueline C. Castilhos^6^, Claudio Ciofi^7^, Nancy N. FitzSimmons^8^, Michael P. Jensen^9^, Angela Formia^7,10^, Colin J. Limpus^11^, Chiara Natali^7^, Luciano S. Soares^12^, Benoit de Thoisy^13^, Scott D. Whiting^14^, Sandro L. Bonatto^2*^

† Sibelle Torres Vilaça and Anelise Torres Hahn are joint first authors

^1^ Department of Life Sciences and Biotechnology, University of Ferrara, Ferrara, Italy

^2^ Escola de Ciências da Saúde e da Vida, Pontifícia Universidade Católica do Rio Grande do Sul, Av. Ipiranga, 668, 90619-900 Porto Alegre, RS, Brazil

^3^ Liberal Studies, New York University, New York, USA

^4^ Unidad Académica Mazatlán, Instituto de Ciencias del Mar y Limnología, Universidad Nacional Autónoma de México, Sinaloa, México

^5^ Hawaiʻi Institute of Marine Biology, University of Hawaiʻi, Kaneohe, Hawaiʻi, USA

^6^ Fundação Projeto Tamar, Sergipe, Brazil

^7^ Department of Biology, University of Florence, Sesto Fiorentino (FI), Italy

^8^ Australian Rivers Institute, Griffith University, Australia

^9^ Department of Chemistry and Bioscience, Section of Biology and Environmental Science, Aalborg University, Denmark.

^10^ Wildlife Conservation Society, Gulf of Guinea Sea Turtle Program, Libreville, Gabon

^11^ Department of Environment and Resource Management, Brisbane, QLD, Australia

^12^Archie Carr Center for Sea Turtle Research, University of Florida, Gainesville, FL, USA and Florida Fish and Wildlife Conservation Commission, Fish and Wildlife Research Institute, Saint Petersburg, FL, USA.

^13^ Kwata NGO, Cayenne, French Guiana

^14^ Department of Biodiversity, Conservation and Attractions, WA, Australia

* Corresponding author: Sandro L. Bonatto, [slbonatto@pucrs.br](mailto:slbonatto@pucrs.br)

**Supplementary Materials**


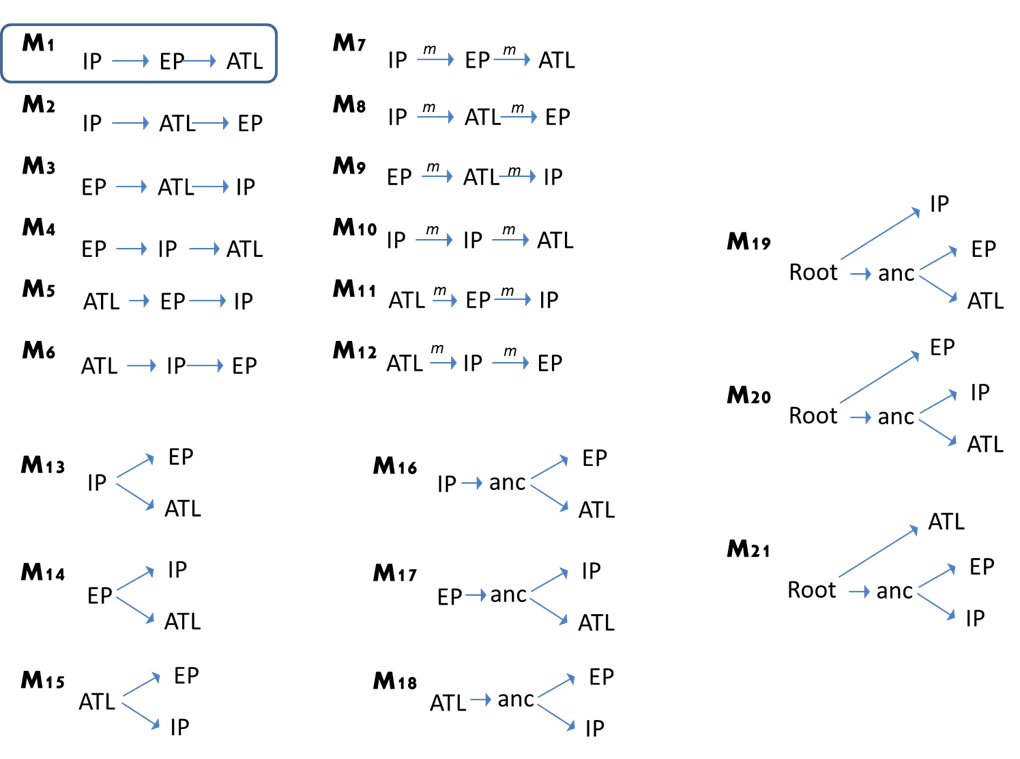
Fig. S1. Scenarios tested with Migrate-n with the microsatellite data. Arrows represent divergence between populations, except for those with *”m”*, that are unidirectional gene flow. **M_1_ was the best model as estimated by Bayes Factor (model probability =1)**. IP = IN/IWP


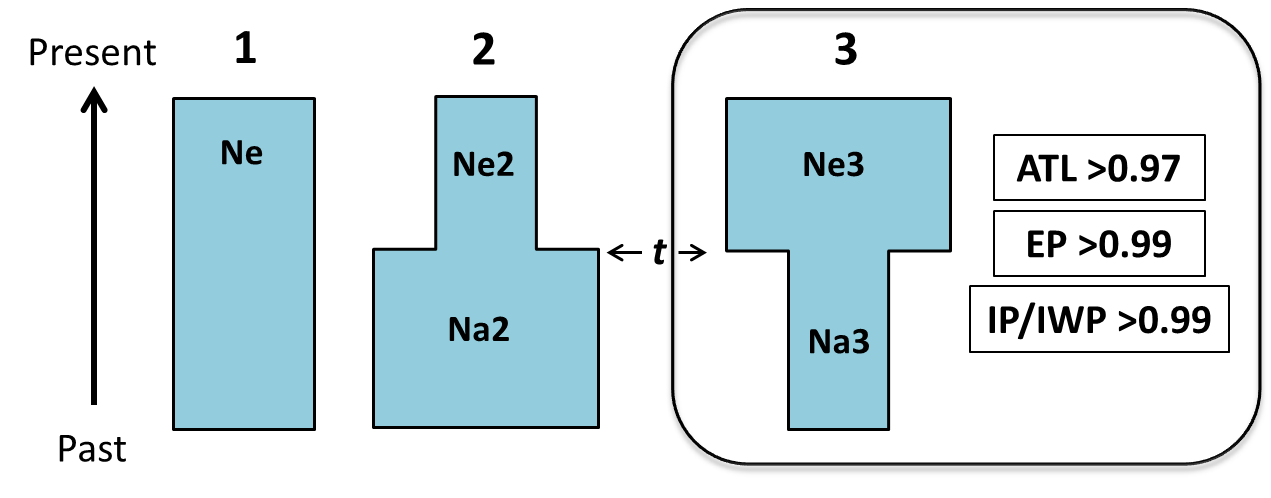


Fig. S2. Scenarios tested with DIYABC with the microsatellite data, as follow: Scenario 1 is stable population, scenario 2 is population reduction, and scenario 3 is populations expansion. NeX and NaX are effective population sizes, and *t* the time of population size change. Priors and posterior values for each parameter are in Tables S4 and 3, respectively. **Outlined is the best scenario for each ocean region with their posterior probabilities.**


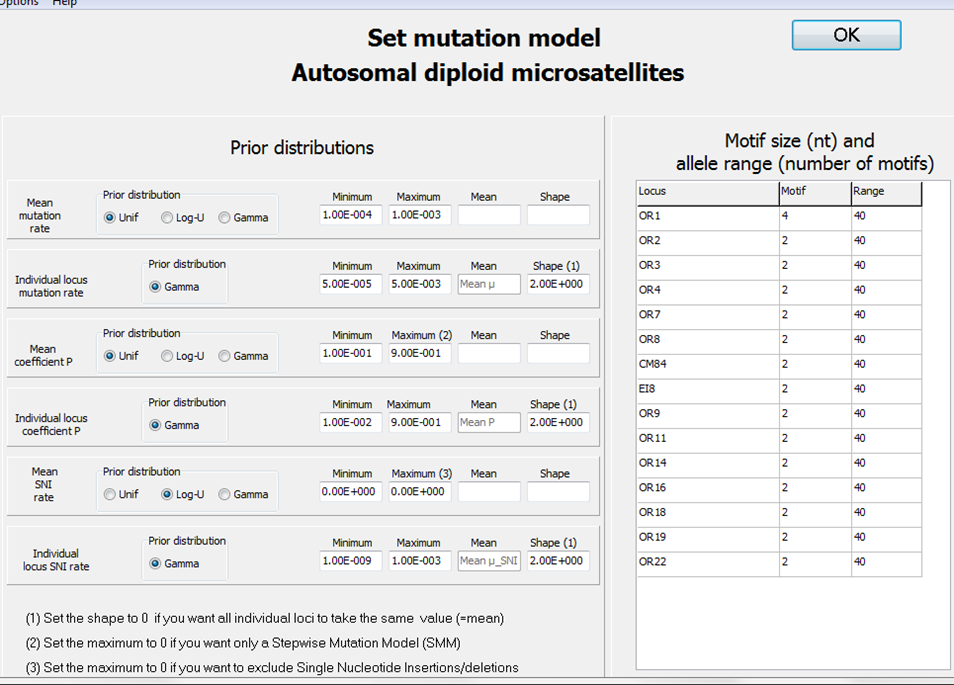


Fig. S3. Priors distributions for the mutational model use with DIYABC.


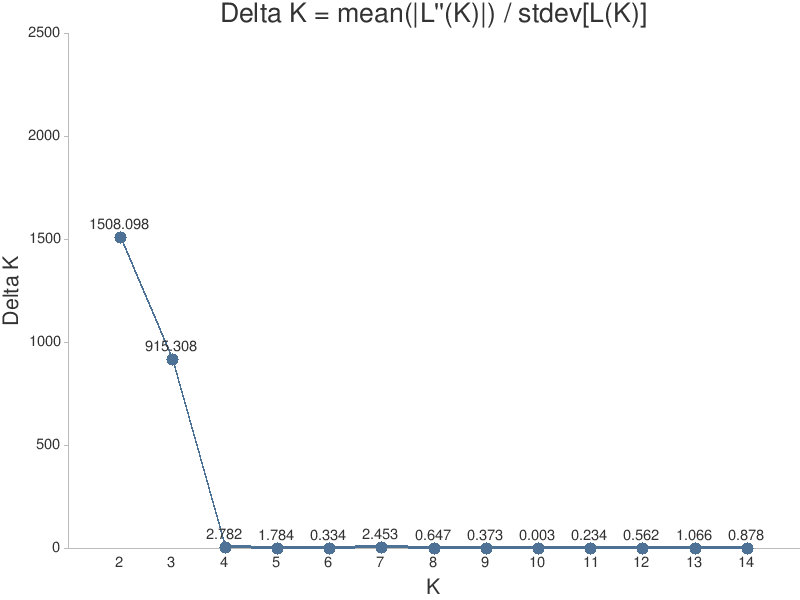

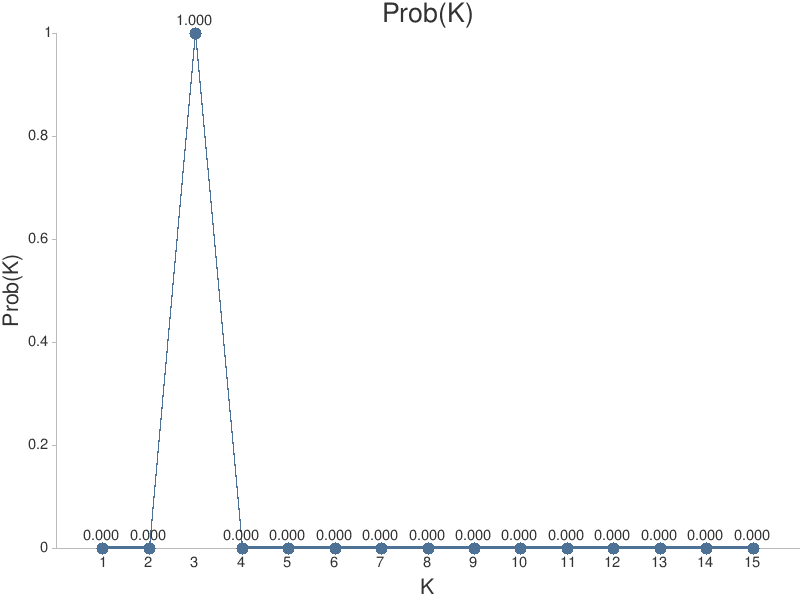


**B**

**A**

Fig. S4. A) ∆K calculated by the Evanno method using CLUMPAK. B) Plot of probability by K graph.


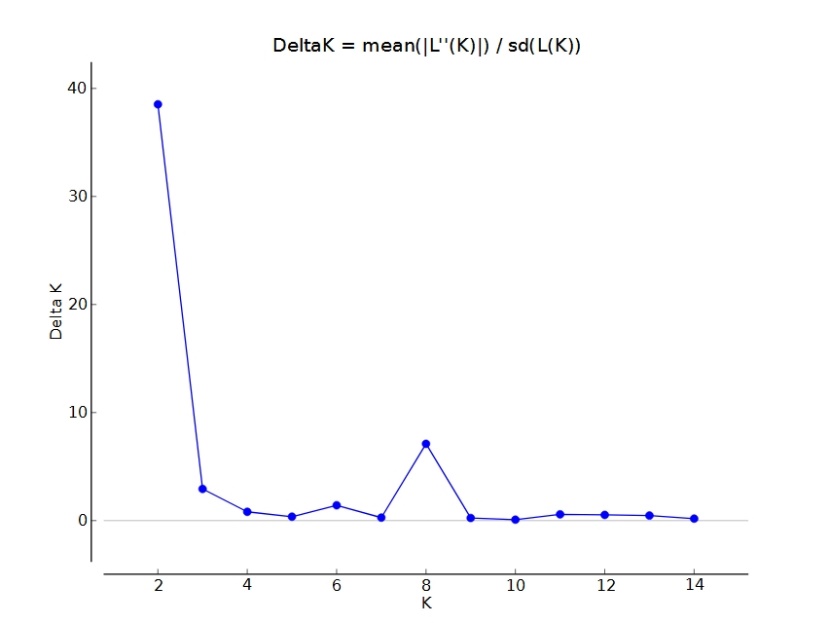

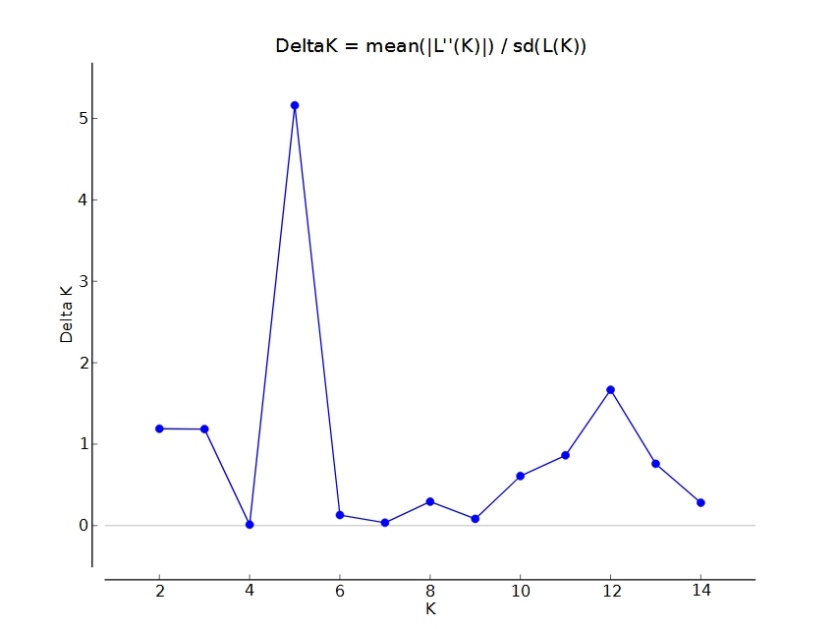


**B**

**A**


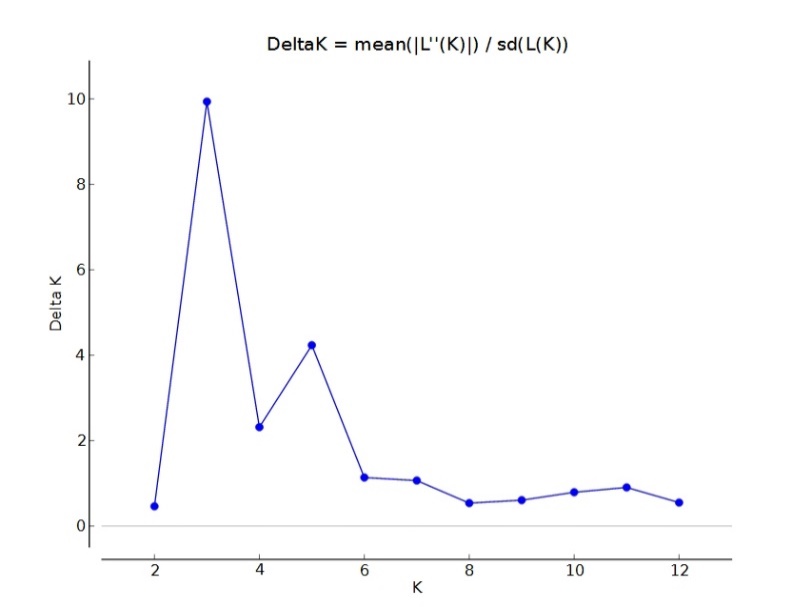


**C**

Fig. S5. Results for ∆K calculated by the Evanno method using CLUMPAK per ocean region. A) Atlantic. B) IP/IWP. C) EP.

**A**

**B**

**C**

Fig. S6: Structure plot results per oceanic region. Only the best K is shown A) Atlantic K=5. B) Indo-Pacific/Indo-West Pacific K=2. C) East Pacific K=3


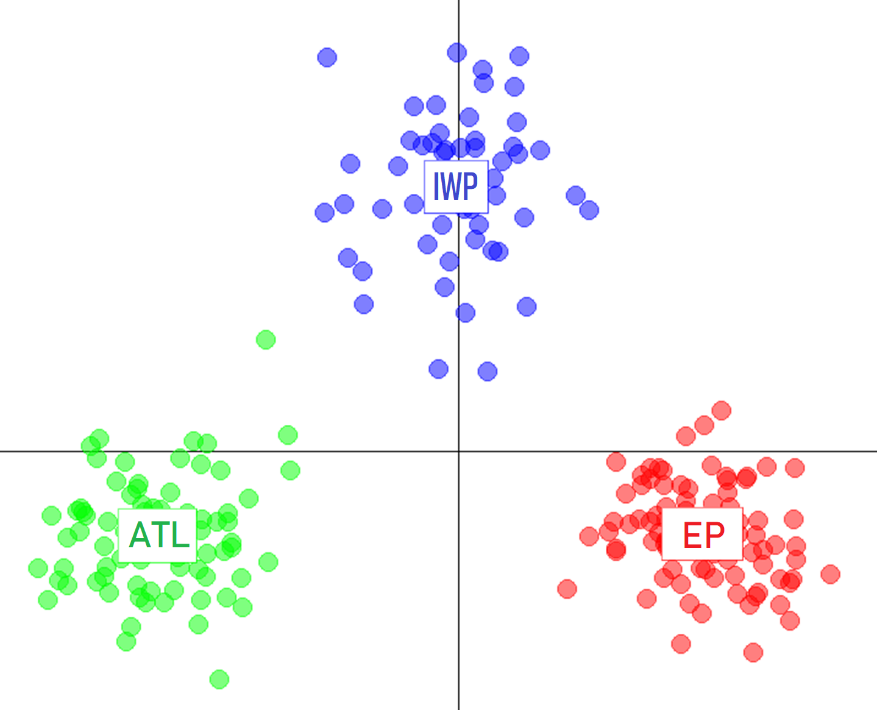


Fig. S7. DAPC results based on individuals' prior grouping into the three ocean regions (ATL, EP, IWP).


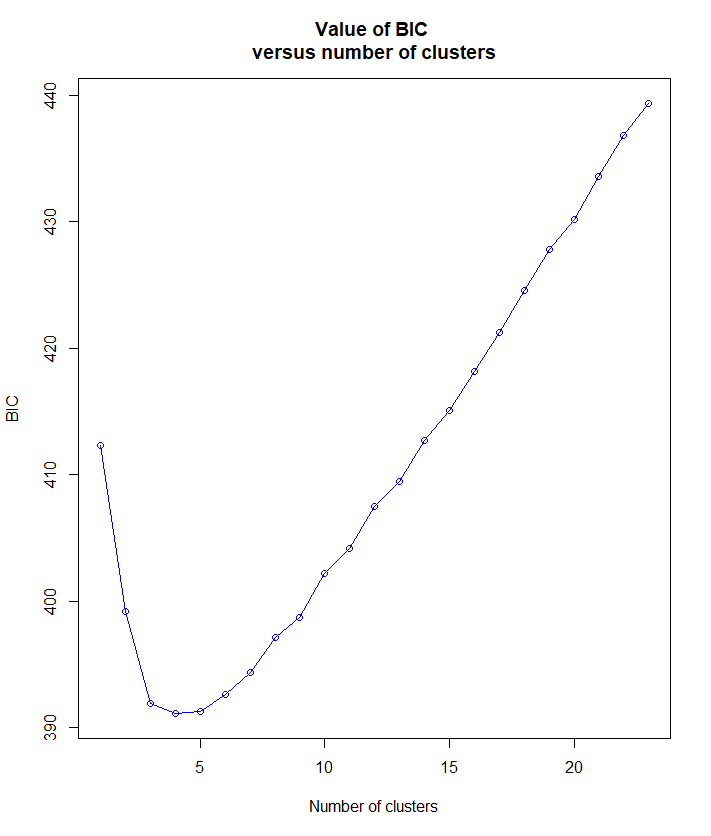


Fig. S8. Graph of Bayesian Information Criterion (BIC) values for increasing values of K

.


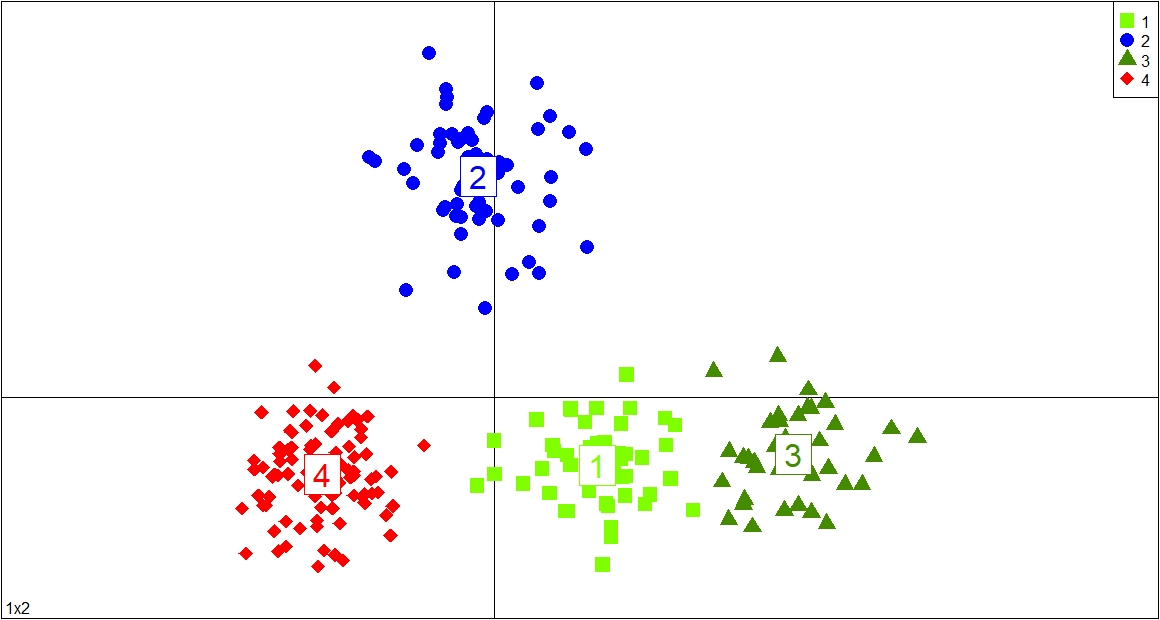


**A**

**B**

Fig. S9. (A) DAPC results with K=4 without prior information. Numbers are the genetic clusters (B) Same results, but individuals were identified by their sampling location. Names in the legend as in Fig. 4.
