# Supplementary material for: G﻿lobal phylogeography of ridley sea turtles (Lepidochelys spp.): evolution, demography, connectivity, and conservation
Source: Conserv Genet. 2022 Aug 29;23(6):995–1010. doi: 10.1007/s10592-022-01465-3 (PMC9659502; doi:10.1007/s10592-022-01465-3)
Supplement: Supplementary file 3 — Supplementary Material 3 [file 10592_2022_1465_MOESM3_ESM.docx]

**Global phylogeography of ridley sea turtles (*Lepidochelys* spp.): Evolution, demography, connectivity, and conservation**

Sibelle Torres Vilaça, Anelise Torres Hahn, Eugenia Naro-Maciel, F. Alberto Abreu-Grobois, Brian W. Bowen, Jaqueline C. Castilhos, Claudio Ciofi, Nancy N. FitzSimmons, Michael P. Jensen, Angela Formia, Colin J. Limpus, Chiara Natali, Luciano S. Soares, Benoit de Thoisy, Scott D. Whiting, Sandro L. Bonatto

Supplementary Text 1

*Estimates of divergence dates using BEAST*

To estimate divergence dates, we employed two calibration points. First, a uniform distribution for the split between *Lepidochelys* and *Caretta* was used and set at 12 - 20 Mya (Duchene *et al.*, 2012). Secondly, the divergence between *L. olivacea* and *L. kempii* was set as an exponential distribution in agreement with fossil evidence suggesting these two species diverged at least 4.5 - 5 Mya (Dodd and Morgan, 1992). The divergence between the two *Lepidochelys* species is based on an *L. kempii* fossil found in Florida and indirectly dated at 4.5 - 5 million years old (Dodd and Morgan, 1992), which should be thus considered as the minimum speciation time. We set the divergence to a mean of 0.5 with a 4.5 offset, which covered an interval between 4.5 - 6.3 Mya with a mean of 5.0 and a median of 4.9 Mya. We used a Coalescent Constant prior to reflect that most haplotypes will follow coalescent events, since most events will be within-species coalescence. A strict clock with the Generalized Time Reversible (GTR) model using four gamma categories was used. Each run took 500 million Markov Chain Monte Carlo (MCMC) iterations sampled every 10,000 steps after a 10% initial burn-in. We checked that all parameters had an effective population size > 200 in Tracer v1.7.1 (Rambaut *et al.*, 2018). Trees were annotated using TreeAnnotator and visualized in Figtree v1.4.4 (Rambaut, 2009).

*Testing of colonization models and estimates of demographic parameters with Migrate-n*

To estimate demographic parameters within regions, we used Migrate-n v4.4.0 (Beerli and Felsenstein, 2001; Beerli and Palczewski, 2010) with a Bayesian approach. The estimated mutation-scaled parameters were theta (Θ = 4*N_e_µ*, where *N_e_* is the effective population size and *µ* is the neutral mutation rate per site per generation) and the immigration rate (M = m/*µ*). Priors for theta and M were set as an exponential distribution from 0.001 to 200 and 0 to 300, respectively. *F*_ST_ was specified as a starting value for estimating M and Θ. Markov chain settings included four heated chains with the default temperatures of 1,000,000.00; 3.00; 1.50; and 1.00, with 20,000 recorded steps every 100 steps and 50,000 steps as burn-in. To scale our results for *N_e_* and the number of immigrants per generation, we employed a mutation rate per site per generation estimated for green sea turtles (*Chelonia mydas*) as 5.7 × 10^−4^ (Fitzsimmons, 1998). Model comparison was calculated using the bf.py python script from Migrate-n package that use the marginal likelihoods to estimate the Bayes Factors and model probability.

Supplementary Text 2

*Microsatellite genotyping*

Nuclear DNA variation was assayed for 285 global *L. olivacea* samples using fifteen microsatellite loci (Table 1).Of these, six were from Aggarwal et al. (2004), seven from Aggarwal et al. (2008), and two from FitzSimmons, Moritz, & Moore (1995) (Table S2). Forward primers were 5’ tailed with the M13 sequence and combined with a fluorescent M13 primer (FAM, NED, and HEX) (Boutin-Ganache *et al.*, 2001). Amplifications were carried out in a final volume of 10 µL with the following reaction mix: ~40ng of Genomic DNA, 1 U Taq DNA polymerase (Invitrogen), 100 µM of dNTPs, 1X PCR Buffer (Invitrogen), 1. 5mM MgCl_2_, 0.0083 µM of M13 tailed forward primer, 0.2 µM of reverse primer and 0.16 µM of M13 fluorescent primer. PCR conditions were: initial denaturation at 94 ^o^C for 4 minutes, 29 cycles at 94 ^o^C for 30 seconds, primer-specific annealing temperature (Table S2) for 40 seconds, extension at 72 ^o^C for 1 minute and 30 seconds, and final extension at 72 ^o^C for 10 minutes. PCR products were genotyped on a MegaBACE 1000 using the ET-ROX 550 size standard (GE Healthcare) and Genetic Profiler v2.2 (GE Healthcare) software. All microsatellite genotyping was conducted in Bonatto’s lab in Porto Alegre, Brazil, to avoid interlaboratory replication problems (Moran *et al.*, 2006).

References

Aggarwal RK, Lalremruata A, Velavan TP, Pavani Sowjanya A, Singh L (2008). Development and characterization of ten novel microsatellite markers from olive ridley sea turtle (Lepidochelys olivacea). *Conserv Genet* **9**: 981–984.

Aggarwal RK, Velavan TP, Udaykumar D, Hendre PS, Shanker K, Choudhury BC, *et al.* (2004). Development and characterization of novel microsatellite markers from the olive ridley sea turtle (Lepidochelys olivacea). *Mol Ecol Notes* **4**: 77–79.

Beerli P, Felsenstein J (2001). Maximum likelihood estimation of a migration matrix and effective population sizes in n subpopulations by using a coalescent approach. *Proc Natl Acad Sci U S A* **98**: 4563–4568.

Beerli P, Palczewski M (2010). Unified framework to evaluate panmixia and migration direction among multiple sampling locations. *Genetics* **185**: 313–326.

Boutin-Ganache I, Raposo M, Raymond M, Deschepper CF (2001). M13-tailed primers improve the readability and usability of microsatellite analyses performed with two different allele-sizing methods. *Biotechniques*.

Dodd CK, Morgan GS (1992). Fossil sea turtles from the early Pliocene Bone Valley Formation, central Florida. *J Herpetol* **26**: 1–8.

Duchene S, Frey A, Alfaro-Núñez A, Dutton PH, Thomas M, Morin PA (2012). Marine turtle mitogenome phylogenetics and evolution. *Mol Phylogenet Evol* **65**: 241–250.

Fitzsimmons NN (1998). Single paternity of clutches and sperm storage in the promiscuous green turtle (Chelonia mydas). *Mol Ecol* **7**: 575–584.

FitzSimmons NN, Moritz C, Moore SS (1995). Conservation and dynamics of microsatellite loci over 300 million years of marine turtle evolution. *Mol Biol Evol*.

Moran P, Teel DJ, Lahood ES, Drake J, Kalinowski S (2006). Standardising multi-laboratory microsatellite data in Pacific salmon: An historical view of the future. *Ecol Freshw Fish* **15**: 597–605.

Rambaut A (2009). FigTree, a graphical viewer of phylogenetic trees. *Inst Evol Biol Univ Edinburgh*.

Rambaut A, Drummond AJ, Xie D, Baele G, Suchard MA (2018). Posterior summarization in Bayesian phylogenetics using Tracer 1.7. *Syst Biol* **67**: 901–904.
